# Roles of the Chr.9p21.3 *ANRIL* Locus in Regulating Inflammation and Implications for Anti-Inflammatory Drug Target Identification

**DOI:** 10.3389/fcvm.2018.00047

**Published:** 2018-05-18

**Authors:** Ghazal Aarabi, Tanja Zeller, Guido Heydecke, Matthias Munz, Arne Schäfer, Udo Seedorf

**Affiliations:** ^1^Department of Prosthetic Dentistry, Center for Dental and Oral Medicine, University Medical Center Hamburg-Eppendorf, Hamburg, Germany; ^2^Department of General and Interventional Cardiology, University Heart Center Hamburg (UHZ), University Medical Center Hamburg-Eppendorf, Hamburg, Germany; ^3^Deutsches Zentrum für Herz-Kreislauf-Forschung (DZHK), Partner Site Hamburg/Lübeck/Kiel, Hamburg, Germany; ^4^Center of Dento-Maxillo-Facial Medicine, Department of Periodontology and Synoptic Dentistry, Charité – Universitätsmedizin Berlin, corporate member of Freie Universität Berlin, Humboldt-Universität zu Berlin, and Berlin Institute of Health, Berlin, Germany; ^5^Institute for Cardiogenetics, University of Lübeck, Lübeck, Germany; ^6^University Heart Center Lübeck, Lübeck, Germany

**Keywords:** periodontitis, inflammation, ANRIL, 9p21.3, drug target, anti-inflammatory agents, coronary artery disease, CDKN2B-AS1

## Abstract

Periodontitis (PD) is a common gingival infectious disease caused by an over-aggressive inflammatory reaction to dysbiosis of the oral microbiome. The disease induces a profound systemic inflammatory host response, that triggers endothelial dysfunction and pro-thrombosis and thus may aggravate atherosclerotic vascular disease and its clinical complications. Recently, a risk haplotype at the *ANRIL*/*CDKN2B-AS1* locus on chromosome 9p21.3, that is not only associated with coronary artery disease / myocardial infarction (CAD/MI) but also with PD, could be identified by genome-wide association studies. The locus encodes ANRIL - a long non-coding RNA (lncRNA) which, like other lncRNAs, regulates genome methylation via interacting with specific DNA sequences and proteins, such as DNA methyltranferases and polycomb proteins, thereby affecting expression of multiple genes by *cis* and *trans* mechanisms. Here, we describe ANRIL regulated genes and metabolic pathways and discuss implications of the findings for target identification of drugs with potentially anti-inflammatory activity in general.

## Introduction

Periodontitis (PD) is an inflammatory disease that involves the osseous, connective, and epithelial, tissues surrounding the teeth (1). Bacteria attached to the teeth along the gingival margin form a biofilm, which may trigger an immune response in the adjacent gingival tissue. If the biofilm is not removed and persists, it can induce gingivitis characterized by swelling, redness and bleeding (2). If the bacterial biofilm and the accompanying inflammatory reaction migrate apically along the root surface and penetrate into the tooth supporting structures the gingival inflammation becomes PD (3), which exists in two forms, chronic periodontitis (CP) and a more severe, early onset form called aggressive periodontitis (AgP) (4). In the US almost 50% of adults aged 30 years or above have CP, including 30% with moderate and 8.5% with severe PD (5). Compared with CP, AgP is less frequent (prevalence: <0.1%). PD is a complex inflammatory disease, which is influenced considerably by interactions between environmental, lifestyle and genetic factors. Some individuals develop PD at young age, although they have similar lifestyle habits and environmental context compared to individuals who do not develop the disease. Therefore, it is considered that early-age of disease onset often indicates a genetic predisposition (6). The genetic susceptibility to PD has been examined extensively by GWAS (7–10) and seven common variants were identified, three of which met the genome-wide significance thresholds. Of the latter three, one (GLT6D1, glycosyltransferase 6 domain containing 1) is specific for AgP, whereas the other two (SIGLEC5, sialic acid binding Ig like lectin 5; DEFA1A3, defensin alpha 1/alpha 3) are associated with both AgP and CP (8, 10, 11). However, to date no associations that met the genome-wide significance threshold for common and rare alleles could be identified for CP alone. It is considered that these not signnificant findings are caused by the small sample sizes that were employed. Yet, some loci give suggestive evidence for association with PD. This evidence is based on independent replication in samples of the same disease phenotype with sufficient statistical power, independent validation of the associations in samples of different disease manifestations, like AgP and CP, and independent identification through different unbiased systematic approaches. According to these criteria, the following loci in addition to *GLT6D1*, *SIGLEC5* and *DEFA1A3* may currently be considered to be associated with CP and/or AgP: *ANRIL* (antisense noncoding RNA in the INK4 locus), *NPY* (neuropeptide Y), *PF4* (platelet factor 4), *PLG* (plasminogen), *VAMP3* (vesicle associated membrane protein 3) (10, 12–20).

Results obtained from longitudinal epidemiological studies support that CAD and CP are associated with each other (21), although the causative relationship between CAD and CP has remained ambiguous (22). Interestingly, variants at *ANRIL*, *PLG* and *VAMP3* were reported to be associated with periodontal phenotypes and also with CAD [recently reviewed in ref. (23)]. Of these, *ANRIL* is the most significant risk locus of CAD and the association of *ANRIL* with PD was replicated repeatedly. In this narrative review, we summarize recent publications on the impact of this locus on chronic inflammation and to discuss potential approaches and strategies to identify new drug targets related to anti-inflammatory therapies in general.

## The Chr.9p21.3 Risk Region Is Shared Between Periodontitis and CAD/MI and Affects Gene Expression of Multiple Genes in Different Cell Types

The 9p21.3 risk haplotype at *ANRIL/CDKN2B-AS1* had initially been identified by GWAS of CAD (24), and was shortly later identified by Schaefer et al. as one of the first genetic risk factors of AgP (17, 25–27) [see (Table 1) for a comparison of the association statistics of the relevant 9p21.3 lead SNPs related to AgP and coronary heart disease].

**Table 1:** Summary of the Chromosome 9p21.3 Locus Associated with Coronary Artery Disease and Periodontitis.

**SNP**	**OR (AgP)**	**OR (CHD)**	**P (AgP)**	**P (CHD)**	**CI 95% (AgP)**	**CI 95% (CHD)**	**N (AgP)**	**N (CHD)**
rs2891168	1.44	1.42	4.4 E-3	1.1 E-6	1.12–1.86	1.23–1.64	159/736	1,104/736
rs1333042	1.44	1.42	4.8 E-3	1.2 E-6	1.12–1.85	1.23–1.64		
rs1333048	1.48	1.39	2.5 E-3	7.6 E-6	1.15–1.92	1.20–1.60		

Association statistics of tree haplotype tagging SNPs at the relevant chromosome 9p21.3 risk region, multiplicative model adjusted for smoking, diabetes, and gender in a logistic regression model. AgP: aggressive periodontitis (generalized), CHD, coronary heart disease (disease onset <55 years), OR: odds ratio, CI: confidence interval, P: P-value obtained from a Wald test, N: number of cases/controls. Data extracted from ref. (17).

The core risk haplotype of ~50 kb, that is shared between CAD/MI and PD encodes the 3’end of a long ncRNA called “antisense non-coding RNA in the INK4 locus (*ANRIL*)” (also designated CDKN2BAS) (17, 25). Its sequence is oriented antisense relative to cyclin-dependent kinase inhibitor 2B (*CDKN2B*), which is located adjacent to the core CAD/PD region. Together with *CDKN2A*, which is located further upstream of *ANRIL*, this region harbors a hotspot for multiple complex human diseases and traits (28). Adjacent is a tightly linked locus for diabetes (29) which is neither associated with CAD (29) nor PD (17).

Given the extended region of high linkage disequilibrium at the 9p21.3 locus and the large number of transcriptional regulatory elements that are present in the CAD risk region, it is currently not entirely clear whether the risk of CAD and PD is mediated solely by ANRIL or whether its neighbors, CDKN2B and CDKN2A - two well-known tumor suppressor genes involved in cell cycle arrest and malignant transformation in certain cancers (30) - contribute to the mechanism. Knockout mice lacking CDKN2B do not only develop a cancer-related phenotype but also advanced aneurysms, accelerated smooth muscle cell apoptosis and medial arterial thinning (31), suggesting a potential involvement of CDKN2B not only in cancer but also in vascular disease. CAD risk SNP rs1537373 affects CDKN2B expression in human coronary artery smooth muscle cells, aorta and the mammary artery (32), and CDKN2B has been shown to regulate inflammatory cytokine production and the clearance of smooth muscle cell-derived apoptotic bodies during atherosclerosis (33). Miller et al. (32) recently investigated the role of SNP rs1537373 in the expression of *ANRIL*. This variant resides in a large haplotype block of linked variants including the highly replicated CAD SNP, rs4977574 and the CAD and PD lead SNP rs1333049 (17, 34). Although rs1537373 does not affect a known transcription factor binding motif, it is located at a site of accessible chromatin. Allele-specific transcription factor binding and histone H3 lysine 27 acetylation around rs1537373 indicated that the native chromatin structure may be affected by the genotype, which was consistent with the observed *cis* eQTL affecting CDKN2B rather than ANRIL in aortic tissues (32). It appears noteworthy in this context that SNP rs1537373 was earlier demonstrated to be also strongly associated with coronary artery calcification (35). If bone marrow lacking murine Cdkn2a was transplanted to the atherosclerosis prone Ldlr(-/-) mouse model, the Cdkn2a-deficient recipients exhibited accelerated atherosclerosis, a higher number of pro-inflammatory monocytes, and increased monocyte/macrophage proliferation compared to controls (36). Thus besides CDKN2B, also CDKN2A has some plausibility for being involved in the pathogenesis of vascular inflammation [see the review by Hannou et al. (37) for further information].

The location of the core risk haplotype of CAD/MI and PD at the 3’end of *ANRIL* implies that the encoded long ncRNA is a prime functional candidate involved in the risk mediating mechanism(s). *ANRIL* is a lowly expressed gene consisting of 20 exons whose transcripts could be detected in a wide variety of cell-types and tissues, including smooth muscle cells, endothelial cells, and cells of the immune system that are known to be involved in atherogenesis (29, 38, 39). Originally, two splice variants were demonstrated in normal human testis and signals using PCR with primers derived from exons 14–16 were also obtained in a range of other tissues (40). Subsequently, many additional splice variants could be identified in various cell-types (38, 41, 42). ANRIL is subject to a complex pathway of alternative splicing which may differ from tissue to tissue and which may be influenced by the presence of SNPs interfering with the function of splice signals.

ANRIL expression was reported to be tightly linked to the *ANRIL* genotype due to disruption of an inhibitory STAT1 binding site in risk allele carriers (43), which would be expected to impair the IFNγ signaling response. However, results published by Almontashiri et al. argued against an involvement of IFNγ in the mechanism underlying the association of the 9p21.3 genotype with CAD risk (44). The CAD risk allele of SNP rs564398, which is one of the SNPs most strongly correlated with ANRIL expression, was predicted to disrupt a Ras Responsive Element Binding protein (RREB) 1 binding site in the 9p21.3 locus (45, 46). RREB may be involved in up-regulating CDKN2B in a Ras-dependent manner by down-regulating ANRIL. Besides stimulating VSMC senescence, Ras has also been implicated to contribute to atherogenesis by affecting vascular inflammation (47). The local functional influence of variants in the 9p21.3 region on gene expression has been examined by many other studies in a variety of tissues and cells (41, 45, 48–52). The results confirmed that the CAD risk variants in the 9p21.3 region are strongly associated with *ANRIL* expression and also with expression of the adjacent loci (*CDKN2A*, *CDKN2B*), albeit much more moderately. However, there is some inconsistency concerning the direction of the effect. Earlier studies suggested associations between CAD risk variants and lower *ANRIL* expression in vascular smooth muscle cells, whole blood cells and purified peripheral blood T-cells (49, 53, 54). In contrast, the study by Holdt et al. (51), in which specifically the long ANRIL transcript (ENST00000428597) was measured, demonstrated that the CAD risk haplotype was associated with higher *ANRIL* expression in whole blood cells and peripheral blood mononuclear cells. Also Zhao et al. found higher expression of this transcript in transformed beta-lymphocytes collected from genotyped donors who carried the CAD risk variant rs7865618 (55). In the latter study, all CAD risk variants assayed in the study were associated with the same directions of the effects.

In addition to the linear form of ANRIL, there also exists a circular ANRIL RNA form (38). Recently, Holdt et al. (56) showed that circular ANRIL may be athero-protective by regulating rRNA maturation. In their model, pescadillo homologue 1 (PES1, a 60S-preribosomal assembly factor) binds to circular ANRIL, which impairs ribosome biogenesis and exonuclease-mediated pre-rRNA processing. The resulting nucleolar stress induces activation of p53, which triggers apoptosis and inhibits proliferation, thereby preventing the accumulation of vascular smooth muscle cells and foam cells at the sites of the atherosclerotic lesion. The balance between atherogenic linear and athero-protective circular ANRIL may be critical for the impact of ANRIL on disease progression. Conversely, a recently published study came to the opposite conclusion, namely that circular ANRIL may be pro-atherogenic (57). In this study, circular antisense ANRIL was used to investigate the inflammatory response of vascular endothelial cells *in vivo* in a rat model of coronary atherosclerosis which was established by injecting rats on a high fat diet with vitamin D3 (57). Circular antisense ANRIL lowered circular ANRIL in vascular endothelial cells along with the levels of several pro-atherogenic markers (serum cholesterol, triglycerides, LDL, IL-1, IL-6, MMP-9, CRP, cANRIL, Bax, caspase-3) and the rates of endothelial cell apoptosis, while HDL levels and bcl-2 expression were increased. In contrast, induction of circular ANRIL expression promoted atherosclerosis by increasing pro-inflammatory properties in vascular endothelial cells and by raising serum lipid and pro-inflammatory cytokine levels. These results were consistent with the hypothesis, that inhibiting circular ANRIL expression would be anti-inflammatory and would reduce vascular endothelial cell apoptosis, which in turn would protect against atherosclerosis in this animal model.

In earlier studies, it could be demonstrated that the epigenetic silencer polycomb repressive complexes 1 and 2 (PRC1 and PRC2) and PRC-associated activating proteins RYBP and YY1 can bind to ANRIL (58, 59), suggesting that ANRIL may be able to modulate epigenetic regulation of target gene expression in *cis* and *trans*. It could be demonstrated *in vitro* by inducible knock-down approaches in T-Rex 293 HEK cells that silencing of two proximal *ANRIL* transcripts altered expression of *ADIPOR1*, *VAMP3* and *TMEM258* (60) (see Table 2 for a list of genes regulated by *ANRIL*). ADIPOR1 is a high-affinity receptor for globular adiponectin, which is involved, amongst others, in PPARα (peroxisome proliferator activated receptor alpha) and AMPK (AMP-activated protein kinase) signaling (62). PPARα activation could prevent experimentally induced bone-loss in animal studies (63). AMPK and PPARα act as key regulators of glucose and fatty acid metabolism in the liver. Adiponectin levels are inversely correlated with BMI, body fat and severity of CAD (64). Globular adiponectin also increases insulin sensitivity by stimulating cellular glucose uptake via increasing recruitment of glucose transporter 4 (GLUT4) to the plasma membrane and inducing *GLUT4* expression (65). Besides these metabolic roles, adiponectin also has anti-inﬂammatory activity by activating tissue inhibitors of metalloproteinases, IL-10, and by suppressing lipopolysaccharide-activated *TNF* (tumor necrosis factor) expression and phagocytic activity (66, 67). The effect of ANRIL on *VAMP3* expression (Table 2) may be important, because VAMP3 belongs to the VAMP/synaptobrevin family involved in phagocytosis and trafficking of TNF-α-containing secretory vesicles to the cell surface required for TNF-α secretion (68).

**Table 2:** *ANRIL*-Regulated Genes

**Gene**	**Mode**	**Gene description**	**Tissue / cell type**	**Disease**	**Ref.**
*ANRIL*	cis	ANRIL, long ncRNA	PBMC, atherosclerotic plaque	CAD, PD	(39)
*CDKN2A*	cis	Cyclin-dependent kinase Inhibitor 2A, tumor suppressor	ANRIL knock-down in VSMC	Cancer	(45)
*CDKN2B*	cis	Cyclin-dependent kinase Inhibitor 2B, tumor suppressor	ANRIL knock-down in VSMC	Cancer	(45)
*ADIPOR1*	trans	Adiponectin receptor 1, glucose, lipid metabolism	inducible ANRIL knock-down in T-Rex 293 HEK cells	Diabetes, CVD	(13)
*VAMP3*	trans	Vesicle-associated membrane protein 3,IL-6, TNFα secretion	inducible ANRIL knock-down in T-Rex 293 HEK cells	Inflammation / cancer	(13)
*C11ORF10*	trans	TMEM258,N-glycosylation	inducible ANRIL knock-down in T-Rex 293 HEK cells	Unknown	(13)
*DUT*	trans	Deoxyuridine Triphosphatase, nucleotide metabolism	Transformed B cells	Unknown	(55)
*EIF1AY*	trans	Eukaryotic translation initiation factor 1A (Y-chr.), mRNA Translation	Transformed B cells	Unknown	(55)
*CASP14*	trans	Caspase-14,Inflammation, apoptosis	Transformed B cells	Psoriasis	(55)
*ABCA1*	trans	ATP binding cassette transporter A1, sterol transport	Transformed B cells	CAD/MI	(55)
*DHRS9*	trans	Dehydrogenase reductase 9, retinol metabolism	Transformed B cells	Unknown	(55)
*CARD8*	trans	Caspase recruitment domain 8, inflammasome	ANRIL knock-down / over-expression in HUVEC, HepG2 cells	Inflammation	(61)

CAD, coronary artery disease; CVD, cardiovascular disease; HUVEC, human umbilical vein endothelial cells; IL, interleukin; PBMC, peripheral blood mononuclear cells; VSMC, vascular smooth muscle cells

Genome-wide *cis* and *trans* effects of the variants in the 9p21.3 region on gene expression were recently studied by Zhao et al. (55), who employed the SNP-set (Sequence) Kernel Association Test [SKAT, (69)] on genotyped transformed beta-lymphocytes collected from 801 participants from the Genetic Epidemiology Network of Arteriopathy (GENOA) study. The results demonstrated a significant association between the CAD and PD risk variants in the region with the expression of the long linear *ANRIL* transcript containing the coding information of all 20 exons except exon 13. In addition to this *cis*-regulatory effect, several *trans* eQTLs could also be identified (Table 2). The affected genes were *DUT* (Deoxyuridine Triphosphatase also known as UTPase), *EIF1AY* (Eukaryotic Translation Initiation Factor 1A, Y-Linked), *CASP14* (Caspase 14), *ABCA1* (ATP-binding cassette transporter A1), and *DHRS9* (Dehydrogenase/Reductase 9) (Table 2) (55).

The *DUT* gene product is an essential enzyme of nucleotide metabolism, which is required for the hydrolysis of dUTP into dUMP and inorganic pyrophosphate. The enzyme plays an important role in controlling the relative cellular levels of dUTP/dTTP (70). Lack or inhibition of dUTPase result in elevated levels of uracil in the DNA, which triggers DNA repair and may induce the formation of DNA double strand breaks, somatic mutations, and apoptosis (71).

**Figure 1:**
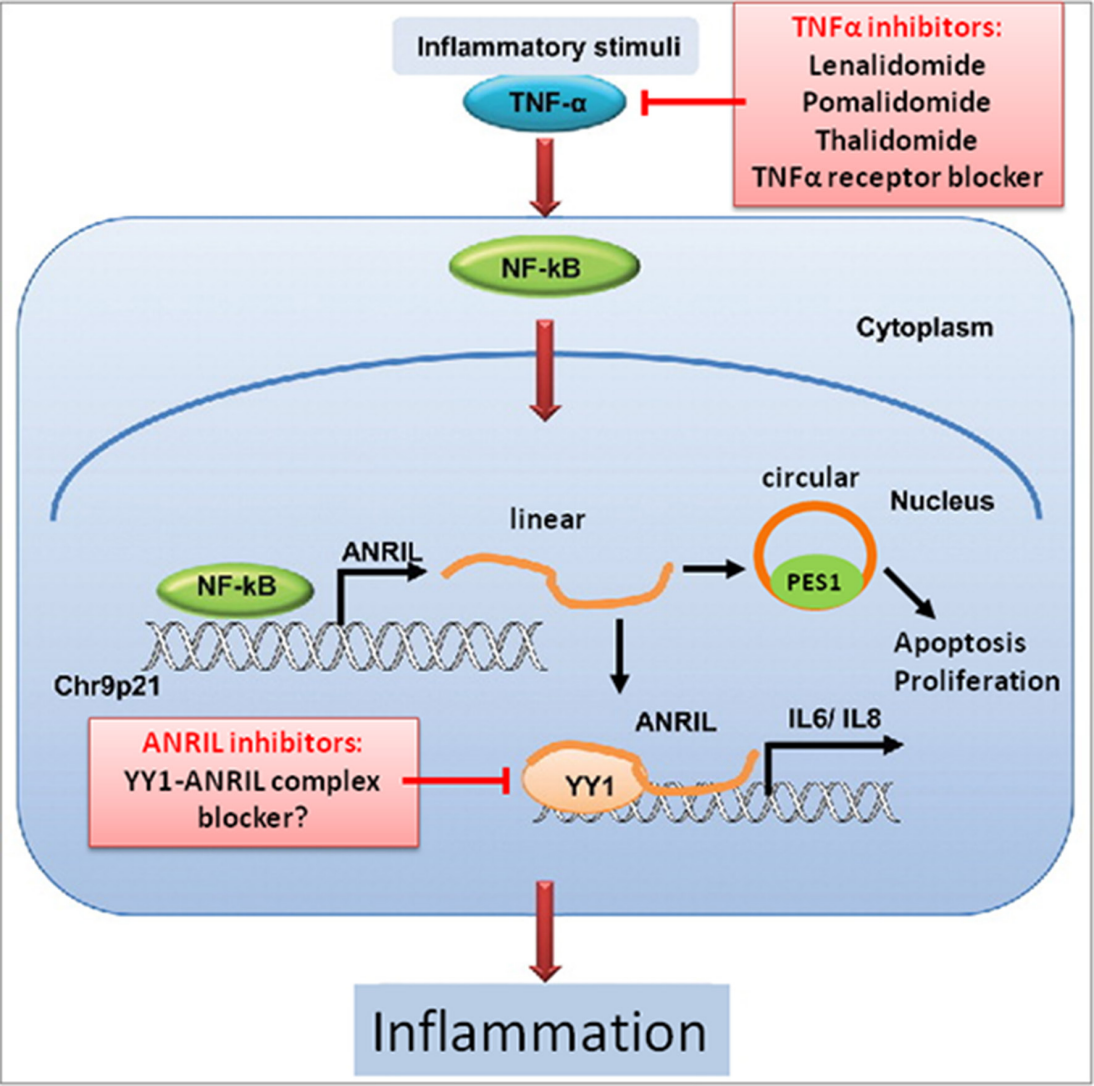
Hypothetical roles of linear and circular ANRIL lncRNA in regulating inflammation and cell survival in human vascular endothelial cells and potential drug targets. TNF-α triggers NF-κB activation, which induces *ANRIL* transcription (66). Linear ANRIL can be converted to circular ANRIL (38). Linear ANRIL interacts with the transcription factor yin yang-1 (YY1) to form a functional complex that binds to and regulates expression of target genes such as IL-6/8. Circular ANRIL interacts with pescadillo homologue 1 (PES1) to form a complex with the pre-ribosomal assembly complex, that impairs ribosome biogenesis, leading to activation of p53 and a subsequent increase in apoptosis and decrease in the proliferative rate (41). This pathway may promote atheroprotection by eliminating over-proliferating cells in atherosclerotic plaques. Neither TNFα nor NF-κB antagonists do seem suitable for wide-spread use in anti-inflammatory therapies of PD or CAD, because of their serious side effects. Since ANRIL is located downstream of TNFα and NF-κB, ANRIL or its downstream targets may be better suited as drug targets to inhibit the pro-inflammatory activities linked to this signaling pathway [modified according to ref. (78)].

*CASP14* is involved in cell apoptosis and is over-expressed in skin, the oral epithelium, bone, heart, and epithelial tumors (72). *EIF1AY* encodes a translation initiation factor which seems to be required for maximal rate of protein biosynthesis (73) and *DHRS9* is involved in retinol and steroid metabolism (74). *ABCA1* plays a well-known role in atherosclerosis (75); but its contribution to PD is unclear. It was proposed that LPS from *P. gingivalis*, which is the most important pathogen involved in PD, may suppress *ABCA1* expression during periodontitis via miRNA-mediated mechanisms (76). To further investigate the potential biological implications of the *trans*-effected genes, Zhao et al. (55) performed gene enrichment analysis on basis of the KEGG Pathway databank. The enriched pathways included “retinol metabolism”, “TGF-β signaling”, and “N-glycan biosynthesis”. Retinol metabolism was at the top of the list of enriched pathways, in which *LRAT* (lecithin retinol acyltransferase),* ADH1* (alcohol dehydrogenase 1), *DHRS9*, *DHRS4L2* (dehydrogenase/reductase 9 and 4 like 2), and CYP26B1 (cytochrome P450 retinoid metabolizing protein) were significantly associated. The importance of TGF-β signaling in the pathogenesis of PD is well-known, since anti-TGF-β antibodies can inhibit the recruitment of leukocytes and the destruction of cartilage and bone at the periodontal lesion sites during periodontitis (77). Another reported downstream target regulated by *ANRIL* is *CARD8* (caspase recruitment domain-containing protein 8) (Table 2) (61). The *CARD8* SNP rs2043211 is significantly associated with ischemic stroke; but its involvement in PD is unclear. The *CARD8* gene product is a component of the inflammasome together with other proteins. ANRIL is induced by pro-inflammatory factors, such as TNFα and IFN-γ, via activation of NF-κB (Figure 1) (78). The transcription factor Yin yang 1 (YY1) can bind to ANRIL and the ANRIL-YY1 complex interacts with the promoter of *IL6/8* to activate *IL6* and *IL8* expression, two cytokines with well established roles in CAD/MI and PD.

Taken together, these findings seem to suggest that *ANRIL* exerts its effects through epigenetic regulation of a great variety of target genes. The common theme seems to be its involvement in expression regulation of genes that play important roles in inflammation, immunity, cell apoptosis and survival, cell proliferation, and metabolism. Many of the reported *trans* regulated genes clearly have plausible roles in CAD and PD as well. Nevertheless, at this stage, we find it premature to formulate a unifying theory that would be consistent with at least the majority of the findings. Most concerning is the apparent complete lack of replication of *trans* regulated genes between the published studies. The reasons for this striking inconsistency may have something to do with the diversity of the experimental approaches and cell-types that have been employed to date. The genome-wide approaches may lack sufficient power to detect some of the differentially expressed genes identified by targeted strategies (55). Antisense approaches are difficult to control due to the complex cell-type specific alternative splicing pathways (38, 41, 42) and findings coming from rodent animal models may not be relevant for humans, since rodent and human ANRIL are evolutionary not well conserved and they differ structurally substantially from each other (79).

## Implications of the Chr.9p21.3 *ANRIL* Locus for Drug Target Identification

Zhou et al. (78) showed that *ANRIL* expression is up-regulated via the TNFα/NF-κB signaling pathway under inflammatory stress conditions (Figure 1). Since endothelial cell-specific inhibition of NF-κB protects mice from atherosclerosis (80), and since *ANRIL* is a downstream target of TNFα/NF-κB signaling, targeting TNFα or NF-κB may theoretically be considered to be athero-protective via inhibiting ANRIL-YY1-mediated IL-6/8 production. Several TNFα receptor antagonists (mostly antibodies) have been tested for safety and efficiency for modulating pro-inflammatory cytokine release in the treatment of rheumatoid arthritis (81). However, clinical trials have shown that these receptor antagonists are associated with increased risks of malignancies and serious infections (81). Since *ANRIL* is located downstream of TNFα and NF-κB, it may be better suited as drug target. However, given the important role of *ANRIL* transcripts in controlling cell growth, its expression is likely precisely regulated. Possibly, putative drug targeting options may come to mind from a better understanding of the precise downstream effects of the linear and circular *ANRIL* lncRNAs on expression of genes involved in chronic inflammatory pathways, suggesting that such work has potential to identify new drug targets for anti-inflammatory intervention.

## Author Contributions

GA and US had the initial idea of writing a review and proposed the topic. Moreover, they conducted extensive literature search and created the first draft of the manuscript. TZ, GH, AS and MM integrated the different information and also wrote and submited the manuscript.

## Conflict of Interest Statement

The authors declare that the research was conducted in the absence of any commercial or financial relationships that could be construed as a potential conflict of interest.
